# A systematic review of artificial intelligence impact assessments

**DOI:** 10.1007/s10462-023-10420-8

**Published:** 2023-03-24

**Authors:** Bernd Carsten Stahl, Josephina Antoniou, Nitika Bhalla, Laurence Brooks, Philip Jansen, Blerta Lindqvist, Alexey Kirichenko, Samuel Marchal, Rowena Rodrigues, Nicole Santiago, Zuzanna Warso, David Wright

**Affiliations:** 1 School of Computer Science, University of Nottingham, Nottingham, UK; 2 Centre for Computing and Social Responsibility, De Montfort University, Leicester, UK; 3 School of Sciences, University of Central Lancashire Cyprus, Larnaka, Cyprus; 4 Information School, University of Sheffield, Sheffield, UK; 5 Department of Philosophy, University of Twente, Enschede, The Netherlands; 6 Department of Computer Science, Aalto University, Espoo, Finland; 7 WithSecure, Helsinki, Finland; 8 Trilateral Research, London, UK; 9 Technology Ethics and Policy Consulting, Kansas City, USA; 10 Trilateral Research, Belview Port, Ireland; 11 Open Future Foundation, Warsaw, Poland

**Keywords:** AI, Impact assessment, Systematic review, AI governance

## Abstract

Artificial intelligence (AI) is producing highly beneficial impacts in many domains, from transport to healthcare, from energy distribution to marketing, but it also raises concerns about undesirable ethical and social consequences. AI impact assessments (AI-IAs) are a way of identifying positive and negative impacts early on to safeguard AI’s benefits and avoid its downsides. This article describes the first systematic review of these AI-IAs. Working with a population of 181 documents, the authors identified 38 actual AI-IAs and subjected them to a rigorous qualitative analysis with regard to their purpose, scope, organisational context, expected issues, timeframe, process and methods, transparency and challenges. The review demonstrates some convergence between AI-IAs. It also shows that the field is not yet at the point of full agreement on content, structure and implementation. The article suggests that AI-IAs are best understood as means to stimulate reflection and discussion concerning the social and ethical consequences of AI ecosystems. Based on the analysis of existing AI-IAs, the authors describe a baseline process of implementing AI-IAs that can be implemented by AI developers and vendors and that can be used as a critical yardstick by regulators and external observers to evaluate organisations’ approaches to AI.

## Introduction

Artificial intelligence (AI) is expected to revolutionise many aspects of our lives, drive efficiency in organisations, improve processes and make better use of resources. Its significant potential economic, social and health (Iqbal et al. 2016; Topol 2019) benefits are, however, counterbalanced by potential disadvantages (Whitby 1991). The Covid-19 pandemic has provided many examples of benefits as well as pitfalls of AI use to address key social challenges (Sipior 2020; Peng et al. 2022). There are concerns about consequences for individuals, not only, for example, when biased systems promote unfair discrimination (Access Now Policy Team 2018) and affect access to social services (Stone et al. 2016), as well as consequences for groups and society, for example, differential profiling and treatment of groups (Persson 2016), political interference (Muller 2020) or when AI leads to concentration of wealth and power (Zuboff 2019), thus exacerbating existing inequalities.

The discussion of how benefits and disadvantages of AI can be understood and balanced covers a range of stakeholders and disciplines. Proposals for proactively addressing possible problems range from ethical guidelines (Jobin et al. 2019) and codes and professionalism (Mittelstadt 2019) to organisational risk management (Clarke 2019a), promotion of explainability (Gunning et al. 2019; Minh et al. 2021) regulatory actions (Clarke 2019b), the strengthening of human rights (Access Now 2018; Council of Europe 2019) and the creation of new institutions (Erdélyi and Goldsmith 2018; Wallach and Marchant 2019). These different responses to negative ethical and human rights consequences of AI need to be seen in conjunction. It is unlikely that any one of them individually will be able to overcome these issues, but collectively they promise ways of understanding and engaging with these issues. There are frequent references to ‘AI ecosystems’, in particular, in the policy-oriented literature (Expert Group on Liability and New Technologies 2019; OECD 2019; UNESCO 2020) that indicate a realisation that a holistic approach will be required.

However, even when using a holistic approach, the question of a suitable starting point remains. When a new AI system transitions from the conceptual stage to design, development and deployment, its technical features, organisational and societal uses become increasingly clear, which then calls for critical reflection of the balance between benefits and downsides. One way to understand possible problems early in the system life cycle and put in place appropriate mitigation measures is to undertake impact assessments for AI. Impact assessments are not a new idea and have a long history in the form of social impact assessment (Becker 2001), environmental impact assessment (Hartley and Wood 2005), human rights impact assessments (Mantelero 2018) as well as more topic-specific impact assessments such as privacy impact assessments (Information Commissioner’s Office 2009; CNIL 2015), data protection impact assessments (Ivanova 2020) and ethics impact assessments (CEN-CENELEC 2017).

An early example of the application of specific impact assessments geared towards AI was provided by the Ada Lovelace Institute (2022). This test took place within the context of the UK’s National Health Service (NHS). The NHS is a large state-run organisation that provides healthcare to all UK residents. It supports research that aims to improve services, reduce cost and support healthcare innovation on a significant scale. The Ada Lovelace Institute’s example focused on the proposed National Medical Imaging Platform from the NHS AI lab. This platform collects NHS data and aims to make it available to private sector and academic researchers which raises interesting questions about the intersection between non-profit and for-profit organisations and resulting questions concerning accountability, liability and distribution of benefits. The project aimed to develop and consolidate an AI-IA process. It involved a literature review, 20 expert interviews and a process development. While this example was still more geared towards the evaluation and assessment of the potential of AI-IAs it gives an indication of what an AI-IA may look like. At the same time, this example shows that AI-IAs are still at an early development stage, thus calling for a systematic review of current approaches.

The idea to apply an impact assessment approach to AI has been proposed in the academic literature (Calvo et al. 2020; Stix 2021) and has found resonance in national policy (UK AI Council 2021) international bodies, such as the European Data Protection Supervisor (EDPS) (2020), the European Fundamental Rights Agency (FRA) (2020) and UNESCO (2020). Such an impact assessment could be supported and/or mandated by a relevant regulatory framework, such as the one proposed by the European Union (European Commission 2021). It could help organisations understand their obligations by providing a basis for their risk assessment of AI (Clarke 2019a) and regulators to ensure that organisations address issues appropriately. It could be a crucial component in the AI ecosystem to ensure that ethical and human rights aspects are taken into consideration and dealt with appropriately.

In this article, we review the current landscape of AI-IAs to understand whether dominant themes and topics can be identified. This allows for the description of a baseline AI-IA that can inform the development of specific AI-IAs as well as organisational, national and international AI policy.

## Methodology

We undertook a systematic review of AI-IAs publicly available as of August 2021. Systematic literature reviews constitute a well-described and well-understood research method (Boell and Cecez-Kecmanovic 2015). Rowe (2014), following Schwarz et al. (2007), suggests that literature reviews can have several goals: to summarise prior research, to critically examine contributions of past research, to explain the results of prior research found within research streams and to clarify alternative views of past research. In our case, we establish a baseline of existing impact assessments to support good practice in future AI-IAs.

There are different ways to undertake a systematic literature review. The main type of input data in which we were interested was text describing existing impact assessment specifically focused on AI. Impact assessments are typically practice-oriented documents that can originate from professional bodies, companies, standardisation bodies and regulatory bodies. There are no comprehensive databases that collect such work. We therefore undertook a multi-pronged approach to identify relevant guidance documents for impact assessments by looking at three bodies of work: (a) a systematic review of the academic literature, (b) general Internet search and (c) snowball and peer searches. The data collection protocol follows precedent on systematic reviews of ethical issues in IT (Stahl et al. 2016) rather than meta-review methods in the biomedical science (Liberati et al. 2009), which is based on methodological assumptions (quantitative data, representativeness of samples etc.) that do not hold for the qualitative data of the AI-IAs in which we are interested.

A crucial question for any systematic review is the definition and limitation of the subject area. From prior general awareness of the literature, we knew that there is a large number of impact assessments that may have some bearing on AI. The following figure provides an overview by indicating three tiers of relevance.

**Fig. 1:**
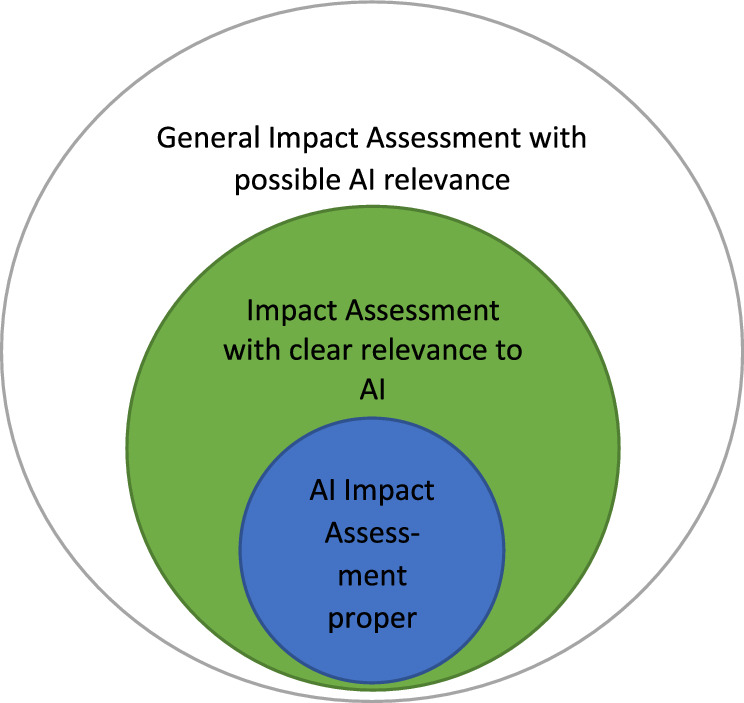
Stratification of documents identified during search using three tiers of relevance

The three tiers are a broad approximation that helped us identify sources to analyse. The actual distribution of sources is more of a continuum from the broadest to very specific impact assessments. Examples of general impact assessments with possible AI relevance include environmental impact assessments (Park and Um 2018; Liu et al. 2021) or human rights impact assessments (Lindblad Kernell et al. 2020). In addition to these general impact assessments, there is a significant number of assessments that touch on one or more aspects that are well established issues in AI such as privacy impact assessments (Clarke 2009; Wright and Friedewald 2013), or technology ethics impact assessment (Wright 2011). We decided for the purpose of manageability of the work but also clarity of analysis to focus on what we call “AI-IA proper” in Fig. 1. We furthermore excluded documents that may serve as part of AI-IA but that have a broader scope, such as the recent IEEE Standard 7000-2021 (IEEE Computer Society 2021), which focuses on systems development more broadly and the CEN/CENELEC CWA 17145 (CEN-CENELEC 2017), which explores ethics assessment for research and innovation more broadly. We realise, however, that some of the broader impact assessments may have a bearing on the practice of assessing AI impact and will therefore return to them in the discussion.

Key questions of relevance to all three types of sources relate to the two core concepts of AI and impact assessment. Our focus is on general applicability and visibility, which is why we used the search terms “artificial intelligence” and “AI”. We added the term “algorithm*” as several examples of AI-IAs use this term, as in “algorithmic impact assessment” (AI Now Institute 2018a; Corriveau 2022; Metcali et al. 2021). We only included documents that proposed impact assessments of AI.

Another methodological choice we made was to focus on AI-IAs guidance documents and exclude documents that only discuss AI-IAs without providing practical guidance on how to implement them. The International Association for Impact Assessment suggests that an impact assessment is “a structured a process for considering the implications, for people and their environment, of proposed actions while there is still an opportunity to modify (or even, if appropriate, abandon) the proposals” (IAIA). Such impact assessments are meant to be applied to decision-making. We therefore only included documents that provided clear evidence of being intended as AI-IAs, e.g., by detailing required processes, scoring criteria or decision relevance. In practice, the dividing line between AI-IAs and texts about them was not always clear, which led the authors to case-by-case discussions and decisions on inclusion/exclusion.

We searched four databases: IEEE, Scopus, ISI and ACM, covering both general academic literature and key databases in the AI/computer science field. These databases were chosen because they include two generic databases covering most academic fields (Scopus, ISI) and computer science (IEEE and ACM) where we expected much of the AI-related literature to be accessible. We focused on papers published since 2015 because the current generation of AI technologies became socially relevant after 2015. The searches of the academic databases and identification of academic papers took place between January and June 2021. Table 1 gives an overview of the searches of the academic literature.

**Table 1:** Search terms for the identification of academic papers

Database	Search term	Further limitations	Number of hits
Web of Science	(TS = (“impact assessment”) OR TI = (“impact assessment”) OR AK = (“impact assessment”)) AND (TI = (“artificial intelligence”) OR TI = (“artificial intelligence”) OR AK = (“artificial intelligence”) OR (TI = (alogrithm*) OR TS = (alogrithm*) OR AK = (alogrithm*))) Indexes = SCI-EXPANDED, SSCI, A&HCI, CPCI-S, CPCI-SSH, ESCI Timespan = Last 5 years	Most recent 5 years	17
IEEE Xplore	((“Document Title”:“impact assessment” OR “Author Keywords”:“impact assessment”) AND (“All Metadata”:“artificial intelligence” OR “All Metadata”:algorithm*))	Filters applied: 2016–2021	16 (3 journals, 13 conference)
Scopus	(TITLE (“impact assessment”) OR KEY (“impact assessment”)) AND (TITLE (“artificial intelligence”) OR KEY (“artificial intelligence”) OR TITLE (algorithm*)) AND (LIMIT-TO (PUBYEAR, 2021) OR LIMIT-TO (PUBYEAR, 2020) OR LIMIT-TO (PUBYEAR, 2019) OR LIMIT-TO (PUBYEAR, 2018) OR LIMIT-TO (PUBYEAR, 2017) OR LIMIT-TO (PUBYEAR, 2016)) AND (LIMIT-TO (SRCTYPE, “j”))	2016–2021	82 documents
ACM DL	[Publication Title: impact assessment] AND [Keywords: impact assessment] AND [Publication Title: artificial intelligence] AND [Keywords: artificial intelligence] AND [Publication Date: (01/01/2016 TO 31/01/2021)]	2016–2021	7 documents

The bibliographic data of all papers was downloaded into a reference management tool (Zotero) for further processing. After removal of duplicates, 122 documents remained.

Realising that most current AI-IAs are practice-oriented and not published in academic outlets, we undertook searches using three search engines (Google, Bing, DuckDuckGo). We used the same search terms as in the academic search: “impact assessment” AND (“artificial intelligence” OR algorithm). In each case, we checked the top 50 hits individually to see whether they contained AI-IAs. We also undertook a set of snowball searches and sought peer input. Snowball searches were triggered by references in any of the other search methods. Realising that there may be AI-IAs in use or in development in organisations that are not (yet) publicly shared, we directly contacted 242 organisations whom we knew to be active in the AI field. We also sent out a request for contributions to eight email lists. We pointed all of these contacts to a web-based survey page where we shared the AI-IAs we had already identified and asked for further suggestions. The email requests for input were sent out between June and August 2021. The web-based survey was closed on 01.09.2021. The collection of documents to be included in the full analysis was concluded on 09.09.2021. No further documents were included after this point, to ensure the consistency and appropriateness of data analysis.

Defining a deadline for data collection and excluding further documents on the basis that they become available after this deadline is a practical necessity for any published survey. It has the disadvantage, however, that more recent contributions to the body of work are not captured and the analysis may not be fully up to date. To some degree this is unavoidable in the traditional academic journal publishing exercise. In our case, however, as we undertook the analysis in the very fast-moving field of AI, this constitutes a more significant concern. We therefore decided to retain our cut-off date for inclusion of documents in the analysis but to reflect on more recent developments that occurred during the review process of the paper (until October 2022) in the Sect. 4.

**Fig. 2:**
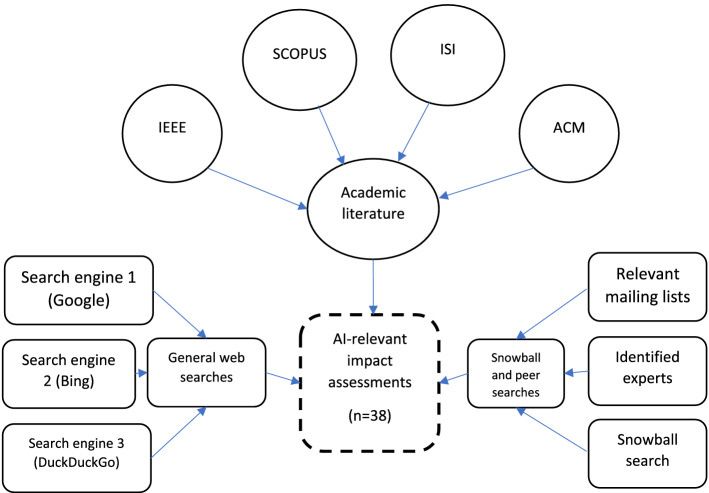
graphical representation of the methodology employed to systematically identify AI-IAs

Figure 2 represents the logic of our method of identifying AI-IAs.

The method of identifying documents as described in Fig. 1 led to the identification of 181 unique documents, after duplicates were removed, to be included in our initial analysis. This sample then underwent a check using the exclusion criteria described above. The application of the exclusion criteria led to the exclusion of approximately ¾ of the sample. In most cases, they were excluded because they used AI in other types of impact assessment, e.g., environmental impact assessment or because they discussed AI-IAs but did not provide practical guidance on how to undertake them. The remaining 43 documents were included in the analysis described below. During the analysis, another five documents were excluded, as more detailed reading revealed that they fell under the exclusion criteria. Initial decisions about inclusion and exclusion were made by the individual authors who were responsible for identifying documents from a specific source (database or search engine). Where the application of the criteria was not straightforward the cases were discussed by the authors to ensure consistency of selection. In cases where no unanimous view was achieved, the default position was to include the source document.

**Fig. 3:**
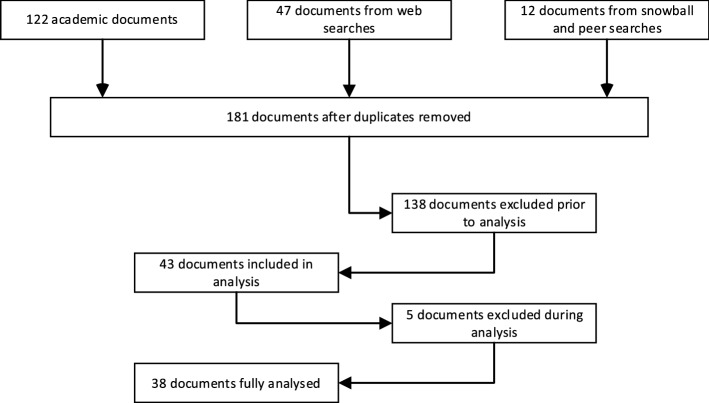
Overview of sample, inclusion and exclusion, following (Liberati et al. 2009)

The final set of 38 documents^1^ that fulfilled our criteria of representing AI-IAs turned out to be highly heterogenous. They included short blog posts as well as elaborate documents. Many were presented as separate files in pdf formats, but some were websites, online surveys or spreadsheets containing evaluation criteria. Some had undergone peer review and were published in academic journals, but most were published on the websites of the organisations that had compiled them. We found IA-AIs originating from academic institutions (16), public sector bodies including regulators (9), standardisation and professional bodies (3), civil society organisations (5) and companies or industry bodies (5). However, these boundaries are not clearly drawn with authorship and ownership of the documents often transcending boundaries. The heterogenous nature of the documents meant that the application of inclusion and exclusion criteria in many cases required deliberation that led to individual judgement calls (Fig. 3).

**Fig. 4:**
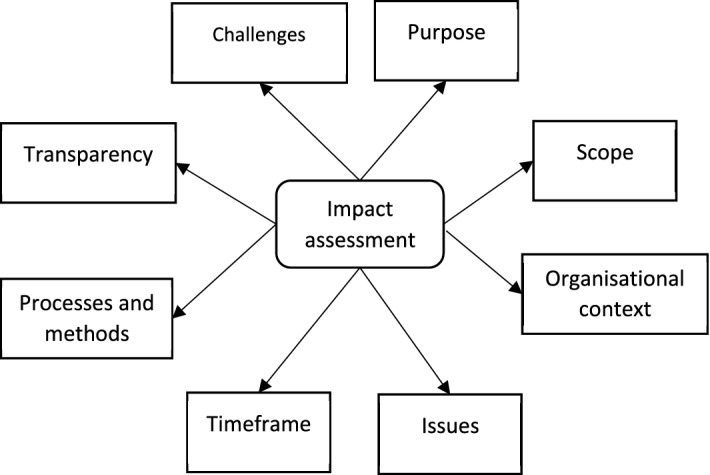
Main analysis topics

The analysis of the AI-IAs was undertaken collectively using the qualitative data analysis software tool NVivo Server version 11. To ensure consistency of analysis, we constructed an analysis framework using thematic analysis principles (Aronson 1995; Braun and Clarke 2006). We started with a set of top-level analysis nodes that were defined according to a general view of likely content of an impact assessment. We hypothesised that an impact assessment could plausibly include the components listed in figure 4.

This figure embodies our assumptions about AI-IAs as follows: We assumed that they would state a purpose for an IA. They could specify their scope and organisational context in which they are undertaken. We expected to find a description of the issues they are likely to face and the timeframe in which the AI-IA is to be undertaken. We assumed that there would be a specification of processes and methods used as well as a sanction for failure to do the AI-IA. We expected there to be a reference to how transparent the AI-IA itself would need to be and a general description of challenges that can arise during the AI-IA.

These eight concepts constituted the starting point for our analysis and thus the main nodes of analysis. The analysis was based on the principle of thematic analysis (Aronson 1995; Braun and Clarke 2006), which is a well-established type of qualitative data analysis(Miles and Huberman 1994). To ensure that the analysis process was open to the identification of new insights and to allow us to show particular areas of interest, we allowed the creation of sub-nodes where these represented either important concepts or captured frequently named topics. For example, we created nodes on “benefits” or “motivation” as sub-nodes under “purpose” or “data protection”, “human rights”, “safety” or “ethics” as sub-nodes under “issues”. These sub-nodes were created following the proposal of one or more coders during regular team meetings and data sessions.

A pilot data analysis was undertaken on two high-profile documents that that constitute AI-IAs (AI Now Institute 2018a; AI HLEG 2020). This allowed us to check the original nodes and to ensure inter-coder reliability. The Kappa-coefficient was determined to be between 0.648 and 0.792 in a pairwise comparison between the lead coder and team members. The Kappa coefficient calculation covered all nodes that were in use during pilot coding phase, not just top-level codes. A Kappa coefficient of between 0.40 and 0.75 is seen as a fair-to-good agreement with a Kappa over 0.75 counting as excellent (QSR). Being satisfied that inter-coder reliability was sufficient, the project team met on a 2-weekly basis to discuss findings and agree on the development of the coding scheme on the basis of insights generated during data analysis.

The coding process was done in a distributed manner following the pilot coding process. This means that source documents were distributed among co-authors who then coded their allocated papers. During the coding phase the team met on a fortnightly basis to discuss progress of coding, open questions, and in particular suggested developments of the coding structure. The principle of coding was that we wanted to remain open to insights from the literature and therefore discussed which changes and addition to the coding structure would be required. Team members could propose new codes where they felt that the existing coding structure failed to provide include important aspects of the literature. This openness included the entire coding structure and would have allowed us to amend the baseline codes listed above. However, it turned out that the top-level codes that we defined in advance and that were also used to structure the findings section in this article were of sufficient quality and granularity to capture key insights. The modification of the coding structure therefore focused on the sub-nodes underneath the main nodes. Decisions on new nodes were discussed in the team with the explicit aim of balancing the required level of detail with the manageability of the overall coding exercise. All 38 papers were fully coded, which means that multiple occurrences of an idea would be coded to the same node. This approach has the disadvantage that it may skew the overall findings in the direction of larger documents that strongly emphasise specific points. However, this disadvantage is outweighed by the advantage that the approach shows the overall emphasis on particular issues across the literature. Being aware of the drawbacks of this approach, however, we are careful not to overstate the statistical significance of the coding distribution.

## Findings

The final set of selected 38 documents constitutes a heterogenous mix. Some of the AI-IAs are traditional documents published by individuals. Several of them do not show individual authors but are attributed to organisations or public bodies. Some implement the assessment activities in their presentation or structure, for example, when they are implemented as interactive online tools (Corriveau 2022) or where they point to supplementary material to be used for assessment purposes (UnBias 2018).

**Fig. 5:**
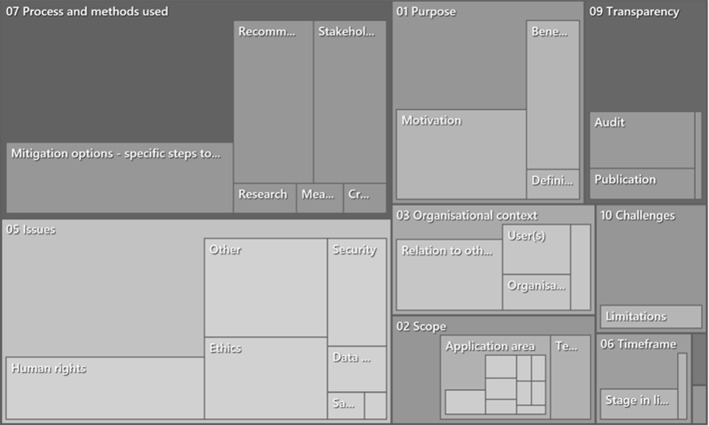
Distribution of most widely used codes during the analysis

The findings of our analysis are structured along the main analysis nodes as indicated in Fig. 4 above and reflected in the structure of this section. In total, we coded 3975 references to 44 nodes with the codes distributed as shown in Fig. 5.

The remainder of this section presents our findings.

### Purpose

Most of the AI-IAs we analysed state their motivation and purpose, which often included a definition of the AI-IA they offer. The motivation for creating an AI-IA can start with current gaps, such as the insufficiency of purely technical assessments (Mantelero 2018), a lack of hard law and absence of established quality assessment methods (Winter et al. 2021). The motivation for the creation of the assessment then covers intended outcomes, such as safeguarding the benefits of AI (AI HLEG 2020), understanding its impacts (Government Accountability Office 2018; IEEE 2020; Raji et al. 2020), assessing system acceptability (AI Now Institute 2018a) and promoting trustworthy AI (Calvo et al. 2020; AI HLEG 2020). These goals are intended to be achieved or promoted by processes that motivate the development of AI-IAs, such as improvements of communication (Gebru et al. 2020), provision of specific methodologies (Brey 2022) that promote good practice, e.g., in data protection (ICO 2020) and more broadly supporting reflection (Gebru et al. 2020).

The AI-IA documents we surveyed suggest that undertaking such an assessment can have numerous benefits that can be split as functional, organisational, individual and social benefits. Functional benefits are those that suggest that undertaking an assessment will lead to better AI systems. AI-IAs aim to achieve this by pointing to known weaknesses, such as biases in machine learning, strengthening accountability and reproducibility and thereby helping researchers and practitioners to select appropriate tools and datasets to mitigate these (Raji et al. 2020). Functional benefits include AI systems that are better tailored to their users’ needs (PWC 2019), that are more responsible (AI HLEG 2020) and thus perceived to be legitimate (Kaminski and Malgieri 2019).

The functional benefits of AI-IAs can easily be translated into benefits for organisations using AI. Making use of AI-IAs is portrayed as a way of improving organisational processes (AI Now Institute 2018a) that support reflection (AI HLEG 2020) and awareness raising (UnBias 2018; Williams 2020) and help identify concerns. The use of assessments promises to strengthen robust governance structures (PWC 2019) that promote organisational oversight (Kaminski and Malgieri 2019), help the organisation define its ethical framework (AI HLEG 2020) and ensure compliance with current as well as future regulation (ICO 2020). Having these mechanisms in place is described as a source of competitive advantage for private companies (PricewaterhouseCoopers 2019; Williams 2020) and good practice in the public sector (UK Governmental Digital Service 2020).

In addition to benefits for organisations, the AI-IAs analysed list benefits for individuals and society. Individuals can benefit by strengthening their rights as data subjects (Williams 2020) and safeguarding their dignity and human rights (Mantelero 2018; Kaminski and Malgieri 2019) and their wellbeing (IEEE 2020). These individual benefits scale on a societal level to the support of fundamental rights more generally (Council of Europe 2019; FRA 2020; Winter et al. 2021). In addition, societal benefits can include the promotion of particular policy goals that can range from furthering the Sustainable Development Goals (ECP Platform for the Information Provision 2019; Williams 2020; AI HLEG 2020) to the more immediate vicinity of AI policy that covers the promotion of responsible innovation (AI HLEG 2020), an increase in trust and avoidance of backlash against new technologies (AI Now Institute 2018a).

There are different views of what constitutes or is conceptualised as an AI-IA. They are frequently described as tools (Williams 2020), which often take the form of self-assessments (Mantelero 2018) that can be used for various purposes, such as audits (ICO 2020) and meeting legal or other requirements (e.g., standards). The description of many AI-IAs makes significant use of the concept of risk management (Oswald 2018; ICO 2020; AI HLEG 2020). AI-IAs are described as facilitating risk estimation (Devitt et al. 2020), risk analysis (Raji et al. 2020), audit (Raji et al. 2020) and mitigation (Brey 2022, p. 1; ICO 2020).

### Scope

The AI-IAs define their scope in different ways. Most of them include reference to the technology covered, the application area or domain or the uses of technology. In many cases, they cover more than one of these. In some cases, this is done as an explicit delimitation of the scope of the document, whereas others explain the scope through examples or case studies.

The technical scope described in the AI-IAs, not surprisingly, has an emphasis on AI (ECP Platform for the Information Provision 2019; Council of Europe 2019). It is worth noting, however, that the terminology is not used uniformly; some documents use terms such as ‘intelligent systems’ (Calvo et al. 2020; IEEE 2020), ‘algorithmic systems’ (Ada Lovelace Institute 2020) or ‘automated decision systems’ (AI Now Institute 2018a). Some cases refer to particular types, notably ‘machine learning’ (PWC 2019; Winter et al. 2021), or relevant features of AI, such as autonomy^53^ or the ability to learn (ECP Platform for the Information Provision 2019; Brey 2022). While this focus is dominant, there are references to broader families of technology, such as emerging (Brey 2022, p. 1) or disruptive (Deloitte Australia 2020) technologies. We also found references pointing beyond particular technologies to the technology ecosystem in which AI is used (Zicari et al. 2021).

The second group of delimitations of scope refers to the application area or domain where AI is to be applied. It is a frequent occurrence for an AI-IA document to highlight the importance of the domain and/or to list various domains calling for particular attention (AI Now Institute 2018a; Corriveau 2022; Government Accountability Office 2018; PWC 2019; Brey 2022; IEEE 2020; Gebru et al. 2020; AI HLEG 2020; Andrade and Kontschieder 2021; Zicari et al. 2021). Among the domains explicitly named, one can find many of those discussed in the media, such as healthcare (Mantelero 2018; Raso et al. 2018; Deloitte Australia 2020; IEEE 2020; Williams 2020; Andrade and Kontschieder 2021; Gardner et al. 2021), finance (Government Accountability Office 2018; Schmitt 2018; Raso et al. 2018), security and law enforcement (Oswald 2018; Mantelero 2018; Kaminski and Malgieri 2019; Deloitte Australia 2020), education (Raso et al. 2018; IEEE 2020; Gardner et al. 2021), transport (Government Accountability Office 2018; Brey 2022, p. 1) and public services (AI Now Institute 2018a; Leslie 2019; Ada Lovelace Institute 2020; IEEE 2020).

A final set of delimitations of the scope points to specific uses of AI that are deemed to be problematic and in need of an AI-IA (Andrade and Kontschieder 2021; Gardner et al. 2021). These include highly contested uses of AI, for example, for surveillance using facial recognition (Deloitte Australia 2020), natural language processing (Andrade and Kontschieder 2021) and cybersecurity (Government Accountability Office 2018).

### Issues

The AI-IAs cover a broad range of issues that can be grouped into the following categories: human rights, ethics, data protection and privacy, security, safety, and environmental impacts. The most frequent topic explicitly referenced is human (or fundamental) rights (AI Now Institute 2018a; Government Accountability Office 2018; Microsoft and Article One 2018; Oswald 2018; Schmitt 2018; Mantelero 2018; Raso et al. 2018; Council of Europe 2019; Kaminski and Malgieri 2019; Deloitte Australia 2020; European Union Agency for Fundamental Rights 2020; ICO 2020; IEEE 2020; Institute for the future of work 2020; Ivanova 2020; UK Governmental Digital Service 2020; Williams 2020; Gebru et al. 2020; AI HLEG 2020; Andrade and Kontschieder 2021; Gardner et al. 2021), with numerous citations to rights as articulated in core international human rights documents (UN General Assembly 1966; European Union 2010). When assessing ethics (AI Now Institute 2018a, b; Oswald 2018; Mantelero 2018; Raso et al. 2018; ECP Platform for the Information Provision 2019; Leslie 2019; PWC 2019; Council of Europe 2019; Calvo et al. 2020; Deloitte Australia 2020; Institute for the future of work 2020; Ivanova 2020; UK Governmental Digital Service 2020; AI HLEG 2020; Zicari et al. 2021; Gardner et al. 2021), the most common ethical issues referenced are bias and non-discrimination, fairness and misuse of personal data. Closely related are issues of data protection and privacy (AI Now Institute 2018a; Oswald 2018; Mantelero 2018; Raso et al. 2018; PricewaterhouseCoopers 2019; PWC 2019; Kaminski and Malgieri 2019; Deloitte Australia 2020; ICO 2020; UK Governmental Digital Service 2020; Williams 2020; Gebru et al. 2020; AI HLEG 2020; Zicari et al. 2021), with about half the AI-IAs referencing legal compliance obligations, most frequently those under the EU General Data Protection Regulation (GDPR 2016). Fewer AI-IA include dedicated discussion on safety (Government Accountability Office 2018; AI Now Institute 2018b; PricewaterhouseCoopers 2019; PWC 2019; Devitt et al. 2020; AI HLEG 2020; Zicari et al. 2021) or security (AI Now Institute 2018a; Government Accountability Office 2018; PricewaterhouseCoopers 2019; PWC 2019; Devitt et al. 2020; ICO 2020; Williams 2020; AI HLEG 2020; Andrade and Kontschieder 2021), the former focused on harm to humans resulting from AI systems and the latter concerned with vulnerabilities of the AI system itself. The final category of issues—environmental impacts (IEEE 2020; UK Governmental Digital Service 2020; AI HLEG 2020) was less frequently included. Additional issues outside of these categories, mentioned only once or twice, include impacts on the labour market and employment (PWC 2019; AI HLEG 2020), accuracy of AI systems (AI HLEG 2020) and impacts on Western democratic systems (Zicari et al. 2021).

### Organisational context

AI-IAs can be embedded in organisational processes and structures in various ways. They can be viewed as part of a broader governance system (Kaminski and Malgieri 2019; ICO 2020) that contributes to AI's responsible governance (PWC 2019). An AI-IA might be embedded in existing processes, including design, assessment and marketing of an AI system (Williams 2020), quality assurance (Raji et al. 2020) or any existing pre-acquisition assessment (AI Now Institute 2018a). But IA-IAs can also be used on their own (Williams 2020). An AI-IA is sometimes carried out by a dedicated team from within the organisation (Raji et al. 2020) or an external body (Mantelero 2018), or both, in those cases where the AI-IA includes a self-assessment phase and an assessment by other stakeholders (AI Now Institute 2018a). If the AI-IA is an internal process, the documents reviewed note the risks of a conflict of interest or a lack of independence of the body/organisation implementing it (Mantelero 2018; Zicari et al. 2021).

The responsibility for the AI is described as falling on the organisations using it, and they are the ones responsible for the IA (AI Now Institute 2018a; ECP Platform for the Information Provision 2019; UK Governmental Digital Service 2020). The documents reviewed suggest that public bodies should be required to conduct self-assessment of AI systems (AI Now Institute 2018a; Council of Europe 2019). At the same time, different aspects of responsibility reside with various actors for ensuring that AI-IA is completed. For example, governments are responsible for setting out procedures for public authorities to carry out an assessment (Council of Europe 2019)and for affected individuals or communities to participate (AI Now Institute 2018a).

AI-IAs have roots in the tradition of impact assessments in different domains, particularly environmental protection, human rights, and privacy (AI Now Institute 2018a; Kaminski and Malgieri 2019). Other types of IAs on which the AI-IAs can draw, overlap and sometimes complement can be broadly grouped into two categories. The first are IAs mainly interested in data: data protection impact assessments (DPIA) (Mantelero 2018; Calvo et al. 2020; European Union Agency for Fundamental Rights 2020; ICO 2020), privacy impacts assessments (PIA) (Mantelero 2018; Kaminski and Malgieri 2019) or surveillance impact assessment (Kaminski and Malgieri 2019). The second category are IAs that focus on societal and ethical impacts. These include ethical impact assessments (Mantelero 2018; Kaminski and Malgieri 2019), societal impact assessments (Mantelero 2018), and equality impact assessments (UK Governmental Digital Service 2020). The assessments differ in terms of their mandatory or voluntary nature (Mantelero 2018). It has been suggested that AI-IAs may be integrated with the DPIA (ICO 2020). In contrast to DPIAs, AI-IA are rarely mandatory (European Union Agency for Fundamental Rights 2020). What distinguishes AI-IAs from other impact assessments is their technology-specificity.

### Timeframe

Regarding the timing of potential AI impacts, only one AI-IA recognized the need to distinguish between short, medium, and long-term risks (Zicari et al. 2021). In terms of the point at which the IA is carried out, if the AI is purchased from another organization, it has been suggested that the IA is implemented before the AI deployment (AI Now Institute 2018a; Institute for the future of work 2020) or, when possible, before its acquisition (AI Now Institute 2018a; Council of Europe 2019). In the case of organizations that design and develop AI, the IA is recommended at the beginning of the project (Corriveau 2022; ECP Platform for the Information Provision 2019). Besides the start of a project, the documents analysed suggest the AI-IA is carried out regularly at several other points of the AI lifecycle (AI Now Institute 2018a; Council of Europe 2019). The documents suggest that AI-IA is revisited and revised at each new phase of AI lifecycle (Council of Europe 2019), when significant changes are introduced (Brey 2022, p. 1), e.g., changes to data collection, storage, analysis or sharing processes (UK Governmental Digital Service 2020) and before the production of the system (Corriveau 2022). It has been suggested that the assessment be renewed at a set time, every couple of years (AI Now Institute 2018a). There is a consensus that AI-IA should be iterative, and the new iterations should be informed by contemporary research and feedback from the AI implementation (European Union Agency for Fundamental Rights 2020; IEEE 2020; AI HLEG 2020).

### Process and methods

Having a recognisable process that allows users to undertake an AI-IA was a criterion for including a document in our sample which ensured they all provided some practical guidance. The structure and detail of the processes covered differ greatly. Most of the IA-IAs describe an explicit structure consisting of phases or steps associated with an AI-IA (AI Now Institute 2018a; ECP Platform for the Information Provision 2019; Ada Lovelace Institute 2020; Brey 2022; Calvo et al. 2020; IEEE 2020). These can start with the determination of what counts as acceptable uses of AI (Deloitte Australia 2020), which can draw upon shared values and principles (Mantelero 2018). This can be part of the preparatory activities of an AI-IA which can also include a definition of benefits expected from the AI (UK Governmental Digital Service 2020) and the need for the impact assessment (ECP Platform for the Information Provision 2019) and the development of skills required to undertake it (ICO 2020). A further preliminary step is the attribution of responsibility for the AI-IA (ECP Platform for the Information Provision 2019; PricewaterhouseCoopers 2019; PWC 2019).

The next steps can start by setting up procedures for documentation and accountability (ECP Platform for the Information Provision 2019) as well as a description of the AI in question (ECP Platform for the Information Provision 2019; Kaminski and Malgieri 2019) and the justification of its use (ECP Platform for the Information Provision 2019). A core component of the AI-IAs is typically a set of questions in the form of a questionnaire or checklist to which the AI-IA seeks responses (Mantelero 2018; Kaminski and Malgieri 2019; Raji et al. 2020; Gebru et al. 2020). These questions are often justified on the basis of existing normative guidance ranging from human rights (Schmitt 2018; Williams 2020; AI HLEG 2020) and existing legislation such as the GDPR, (Kaminski and Malgieri 2019) to lists of ethical issues (Devitt et al. 2020), principles of sustainability (AI Now Institute 2018a; AI HLEG 2020) and responsible innovation (Raji et al. 2020). These questions cover the various issues associated with AI such as data protection (AI Now Institute 2018a; Kaminski and Malgieri 2019; AI HLEG 2020), data quality and representativeness of data (UK Governmental Digital Service 2020), fairness (Schmitt 2018), reproducibility (UK Governmental Digital Service 2020), explainability (UK Governmental Digital Service 2020), transparency and accessibility (AI HLEG 2020). Often, there is a recognition of trade-offs between some of these issues (ICO 2020). Often, these questions lead to a quantitative scoring of issues and risks (Corriveau 2022; ICO 2020) or the determination of key performance indicators. These draw on scientific insights (AI Now Institute 2018a; IEEE 2020; AI HLEG 2020) from various disciplines, such as psychology (Calvo et al. 2020) or foresight analysis (Brey 2022).

A further aspect shared by many of the AI-IAs is the inclusion of stakeholders in the assessment process (AI Now Institute 2018a; Mantelero 2018; Kaminski and Malgieri 2019; IEEE 2020; Raji et al. 2020; AI HLEG 2020). Considerable effort is spent on the identification of suitable stakeholders who are typically expected to cover the relevant areas of expertise of the AI application as well as the groups affected by it. Examples of such stakeholder groups include AI users (Andrade and Kontschieder 2021), external experts (Kaminski and Malgieri 2019; Ada Lovelace Institute 2020), technology providers (ECP Platform for the Information Provision 2019), senior manager (ICO 2020) and civil society more broadly (Kaminski and Malgieri 2019).

Following the identification of issues, most AI-IAs proceed to outline specific steps that can be used to mitigate undesirable consequences of AI (Council of Europe 2019; Brey 2022, p. 1; ICO 2020; Ivanova 2020). There are numerous categories of mitigation measures (ECP Platform for the Information Provision 2019; Kaminski and Malgieri 2019; Andrade and Kontschieder 2021) including technical measures such as de-biasing training data (Ivanova 2020) or code inspections (Ada Lovelace Institute 2020) and organisational measures (PricewaterhouseCoopers 2019; AI HLEG 2020) such as the creation of accountability structures (AI HLEG 2020), documentation (UK Governmental Digital Service 2020), evaluation and monitoring of systems use (UK Governmental Digital Service 2020) but also enabling human interventions (Ivanova 2020). One can find suggestions for inclusion and diversity(AI HLEG 2020), promoting training and education of the workforce (Brey 2022, p. 1; UK Governmental Digital Service 2020; Andrade and Kontschieder 2021), the inclusion of external experts (Kaminski and Malgieri 2019) and the definition of redress mechanisms (AI HLEG 2020). These mitigation measures all suffer, however, from the uncertainty of future occurrences (Deloitte Australia 2020) that can require situation-specific responses (Deloitte Australia 2020) and call for the maintenance of mitigation mechanisms over time (Zicari et al. 2021).

### Transparency

The AI-IA documents share a common standpoint concerning the importance of transparency and communication in AI systems. Transparency means that actions, processes and data are made open to inspection by publishing information about the project in a complete, open, understandable, easily-accessible and free format (UK Governmental Digital Service 2020).

The key is to help humans understand why a particular decision has been made and provide the confidence that the AI model or system has been tested and makes sense. Transparency about how an AI application works gives individuals the opportunity to appreciate the effects of the application on the freedom of action and the room to make decisions (ECP Platform for the Information Provision 2019). In practice, this can mean various things. It may mean that there is access to the source code of an AI application, that to a certain extent, end-users are involved in the design process of the application or that an explanation is provided in general terms about the operation and context of the AI application. Transparency about the use of AI applications may enlarge the individual's autonomy, because it gives the individual the opportunity to relate to, for instance, an automatically made decision (ECP Platform for the Information Provision 2019).

However, limitations in the ability to interpret AI decisions not only is frustrating for end-users and customers, but can also expose an organisation to operational, reputational and financial risks (PWC 2019). To instil trust in AI systems, people must be able to look “under the hood” at their underlying models, explore the data used to train them, expose the reasoning behind each decision and provide coherent explanations to all stakeholders in a timely manner (PWC 2019). Individuals must perceive that they have a reasonable voice in the decision-making process, that the decision-makers have treated them respectfully and that they regard the procedure as fair (Deloitte Australia 2020).

A trustworthy approach is key to enabling ‘responsible competitiveness’, by providing the foundation upon which all those using or affected by AI systems can trust that their design, development and use are lawful, ethical and robust (AI HLEG 2020). A crucial component of achieving trustworthy AI is transparency, which encompasses three elements: (1) traceability, (2) explainability and (3) open communication about the limitations of the AI system (AI HLEG 2020).

It has been suggested that, with every deployment of AI, the organisation look at what is required for transparency and what that means for the design of the technology, the organisation or the people working with the technology (ECP Platform for the Information Provision 2019). For example, companies could be required to publicly disclose information about each automated decision system, including details about its purpose, reach, potential internal use policies or practices, and implementation timeline (AI Now Institute 2018a). The initial disclosure provides a strong foundation for building public trust through appropriate levels of transparency, while subsequent requests can solicit further information or present new evidence, research or other inputs that the agency may not have adequately considered (AI Now Institute 2018a).

Currently, few organisations are explicitly mandated to disclose anything about the systems they have in place or are planning to use. Instead, impacted communities, the public at large and governments are left to rely on what journalists, researchers and public records requests have been able to expose (AI Now Institute 2018a). However, government bodies and external auditors can play a crucial role in enabling open transparency between the AI technology and its users, but robust processes must be in place to carry out the audit effectively. Auditing tools must be explicit and clear about which definitions they evaluate, what those definitions mean and in what ways they are limited (Institute for the future of work 2020). Auditing must fit within a broader approach to evaluate the impact of AI systems on equality. This comprehensive evaluation should include consideration of impacts on equality of opportunity and outcome, and focus companies on making adjustments to mitigate identified adverse impacts (Institute for the future of work 2020). Furthermore, the auditors must live up to an ethical standard themselves in order to enhance fairness and evaluate the impact of the AI system over time.

### Challenges

Assessing the impact of AI raises significant challenges, starting from the variety of AI applications themselves, which makes it more difficult to understand the nature of AI and its consequences and how these are reflected in social norms (Deloitte Australia 2020). For example, assessing the impact of an AI solution may involve the consideration of fairness in terms of the existence of bias, but it may involve trade-offs that render it impossible to be fair to everybody (PWC 2019). However, even though there is continuous demand for more regulation (Calvo et al. 2020), the arguments on the flipside, e.g., that such regulation slows innovation, are increasing. The open nature of AI as a general-purpose technology renders prediction of consequences difficult, which contributes to challenges of governance (Raji et al. 2020).

Assessing an AI system’s impact, considering both ethics and innovation, is an important part of an AI impact assessment, but the impact itself is difficult to model (Calvo et al. 2020), especially because AI-based systems are not static, as usually assumed by traditional impact assessments; instead, they are dynamic as they are adding new data, learning and refining models (Calvo et al. 2020). In addition, to accurately capturing the system itself, assessors must give attention to the way that the system is used in a particular organisation and the structure of any impact assessment procedure such that it does not end up being excessively burdensome and complex (Mantelero 2018). Additionally, defining values as benchmarks in an impact assessment procedure becomes challenging just because of the variety and complexity of such values and the need to tailor them to the specific application (Mantelero 2018). This refined assessment approach may generate additional burden on companies as they may be expected to identify and mitigate every conceivable risk (Andrade and Kontschieder 2021).

## Discussion

Our analysis has shown that there is broad interest in AI-IAs from various quarters. AI-IAs offer a practical approach to the ethical and social issues of AI that is missing from the guideline-centric approach that currently dominates the debate (Stix 2021). Our research suggests that there is a certain level of convergence between AI-IAs. However, the research also shows that a number of open questions remain. We structure this discussion around some key issues: conceptual questions, the impact of AI-IAs, costs and benefits, driving forces behind AI-IAs, framing of AI-IAs and we finish with a brief review of the rapidly changing landscape in which AI-IAs develop.

### Conceptual questions

A first set of questions pertain to concepts and definitions. While AI is broadly discussed and definitions of AI abound, there is no universally accepted and unambiguous definition of AI (Elsevier 2018), which makes it difficult to delineate the exact scope of an AI-IA. This is reflected in the titles of many of the documents reviewed, which use other terms such as ‘algorithm’ or ‘big data’. These other terms do not solve the problem, as they introduce new ambiguities. Exact definitions of terms are usually difficult to agree. In the case of AI-IAs, this lack of a clear definition of the technology to which it refers is problematic for several reasons. A broad definition of the underlying technology may call for a sweeping application of such AI-IAs, which could be prohibitively costly and at the same time not plausible. If, for example, one were to undertake a full impact assessment of all technical systems that are based on or incorporate algorithms, then this would cover most outputs of computer programming, which would be far too broad. A narrow scope, for example, one focusing on particular types of applications of deep learning only, might miss new developments and not capture developments that have significant potential for risk. A further problem of the lack of a clear definition of AI is that it renders a general application of AI-IAs unlikely, as owners and users of AI may justifiably argue that it is not clear which systems exactly are to be subject of such an assessment.

Further conceptual questions arise with regard to the scope and scale of AI-IAs. Some of the documents analysed have a broad scope and ambition whereas others focus on specific applications or issues. Some are predominantly focused on the technology in question whereas others think more broadly in terms of organisational embedding of technology, required capacities by staff to deal with them, etc. This breadth of scope is not problematic per se, but it raises the question about how many AI-IAs are needed. A large number may be useful in catering for many applications, but it has the disadvantage of making it difficult for potential users to understand the landscape and choose the most appropriate AI-IA.

### Impact of AI-IAs

A further fundamental question is whether a particular AI will have an impact at all or an impact that calls for an AI-IA. Any use of an AI will have some impact; otherwise, there would be no point in employing it. However, only when there is reason to believe that an AI is likely to lead to socially or ethically relevant changes does it make sense to consider whether these changes are positive, negative, call for mitigation measures, etc. Impact, in many cases, can be defined rigorously, though what definitions optimally capture the most important aspects in a given use case can be a challenging question. A good example of impact definition is provided by Berk (2017) in the context of the use of machine learning forecasts by a parole board to help inform parole release decisions. The article defines and evaluates the impact of the forecasts through stating and addressing the following three questions: Did the overall proportions of inmates released by the Board change because of the forecasts? Did the forecasts lead to changes in the kinds of inmates the Board released on parole? What impact, if any, did the forecasts have on arrests after individuals were paroled?

Defining impact in such a manner can enable us, in principle, to evaluate it via statistical hypothesis testing. A key challenge in applying a mathematically rigorous method is, of course, the availability of datasets satisfying certain requirements. In the case described by Berk (2017), for example, because the machine learning system was introduced into the Board operations gradually, it was possible to split a large set of parole cases into the treatment group and the comparison group, and the randomness assumption about the composition of the groups appeared plausible. While such datasets may not always be readily available for deployed AI-powered systems, we think that their designers, integrators and operators often have sufficient control for enabling an impact assessment.

The question whether an AI has an impact introduces numerous considerations. One observation from our analysis is that many of the AI systems under discussion are still under development or found in a research setting. In such cases, even the intended outcomes may not be clear, which makes it difficult to determine which impacts to look for. The aim of AI-IAs to deliver technical, individual, organisational and societal benefits makes the determination of relevant impacts challenging. Many of the documents analysed refer to ethical principles or human rights. In some cases, the impacts on these will be possible to capture, as the example of parole decisions indicates. In other cases where impacts are based on broader concepts, such as human dignity or social justice, this will be more challenging.

### Costs and benefits

The issue of measuring impacts leads to questions of trade-offs within AI-IAs as well as the cost–benefit balance of the AI-IA approach as a whole. Trade-offs can be expected in many impact assessments where an aspect deemed desirable leads to consequences that are undesirable. In AI, for example, it is likely that trade-offs will appear between privacy of individuals versus transparency of the AI. Many similar trade-offs are conceivable and should be captured and evaluated by an AI-IA. The cost–benefit balance of the AI-IA approach as a whole is a special type of trade-off. The benefits of an AI-IA not only depend on the identifiability of impacts but also on whether the impact assessment has consequences that support desired impacts. Measuring such impacts will be difficult, if not impossible. This is caused by the potential of long-term impacts which are difficult to measure in the short term and may be impossible to measure or to quantify at all. The costs of undertaking an AI-IA may be easy to measure on an organisational level. However, in addition to the immediate financial costs of undertaking an AI-IA, there may be side effects, such as slowing down the rate of innovation or self-censoring by innovators, which can be counted as costs impossible to measure on a societal level.

Such questions are not confined to AI-IAs, but similarly apply to other types of impact assessment or risk management measures. It is, therefore, important to consider the embedding of AI-IAs in existing structures. Our analysis has shown that many AI-IAs reference other types of impact assessment and it therefore seems reasonable to embed them in established activities, such as due diligence or risk management processes that may already cover environmental or other impact assessments. One important part of the discussion that has the potential to significantly affect the cost–benefit analysis from an organisational point of view is that of sanctions for undertaking (or omitting) AI-IAs. If an organisation could be fined or if its liability threshold were to change because of an AI-IA, this would change its willingness to undertake one. Interestingly, however, our analysis of the existing AI-IAs found little reference to such external sanctions. The majority of the AI-IAs investigated relied on positive messages and the benefits of AI-IAs with little reference to legal or other mandates to undertake them or negative sanctions for failing to do so.

The current landscape of AI-IAs thus retains numerous open questions. While significant efforts have been undertaken in defining and trialling such IAs, there remain a number of concerns. Existing AI-IAs are intended to do good, but it is often not clear who will benefit from them or how competing interests are considered, e.g., when organisational benefits conflict with societal ones. The current landscape furthermore shows the danger of fragmentation. Our sample included 38 AI-IAs and we can expect the number to grow. This leads to problems for the user in choosing an appropriate AI-IA model. More importantly, it makes it difficult to assess who will benefit from applying any individual AI-IA. In addition, the application of AI-IAs is fraught with uncertainty and subjectivity. Many of the aspects of AI-IAs are open to interpretation. Abstract criteria and grading scales are sometimes provided but grading can be highly subjective. There is a trade-off between being generic (and proposing an IA process that can be applied to virtually any use case) and scientific precision, which may be impossible to achieve.

### Driving forces of AI-IAs

The question of costs and benefits of AI-IAs discussed in the previous sub-section links directly to the question why one would undertake such an assessment in the first place. As the analysis of the purposes of AI-IAs in Sect. 3.1 has shown, there is a mix of intentions whose relationship is not always obvious. One way of approaching the mix of motivations is to look at two extreme or ideal typical positions. On the one hand, an AI-IA can be undertaken for purely functional reasons, i.e., in ways that will benefit the organisation. Such an approach would be anchored in cost–benefit analysis and interpret ethical and social concerns arising from AI as a potential threat to the organisation that needs to be addressed to avoid possible damage. On the other hand, an AI-IA might be driven by more altruistic motivations, such as a desire to do the ethically right thing or to uphold human rights.

It is easy to see that these two positions may come into conflict, e.g., where an AI system jeopardises human rights but it does so in ways that have no likely implications for the organisation. Similarly, there may be risk to the organisation which do not involve significant ethical or human rights concerns. In such cases, the reaction to the issue in question would differ significantly, depending on the main driving force motivating the implementation of AI-IA.

While this consideration of ideal typical position is instructive in understanding how AI-IAs can be interpreted from the perspective of the organisation employing them, in practice this distinction between different driving forces is much less clear. Most of the more elaborate documents we analysed have a general introduction setting the scene and providing a rationale for introducing the AI-IA. These typically refer to broader societal aims, including ethical considerations and the need to adhere to human rights. The emphasis on these general justifications differs between different documents but they are generally visible. At the same time, the implementation of broader ethical and human rights aims can be achieved through risk management processes that are based on cost–benefit considerations. Costs and benefits can be purely financial, but they do not have to be. The scope of such considerations depends on the interpretation of the organisation in question. Similarly, a human rights-based can be expressed in terms of costs and benefits which again can but do not have to be financial.

One can thus conclude that the driving forces behind the implementation of an AI-IA can differ broadly, ranging from the purely defensive and egotistical to a broad embrace of the public good. In practice, however, this intention does not seem to be determined or clearly represented by the chosen approach or method.

### Framing of AI-IAs

These concerns lead to a larger one that AI-IAs will be used for what is sometimes called ‘ethics washing’ (Wagner 2018). Several authors have observed that the AI ethics debate is in constant danger of being hijacked by particular interests, most notably the interests of large corporations who have a vested interest in using ethical rhetoric to avoid regulation and deflect scrutiny (Nemitz 2018; Coeckelbergh 2019; Mittelstadt 2019; Findlay and Seah 2020). The use of AI-IAs would be a good tool for such purposes, as it remains within the remit of the organisation to implement and publicise disseminate it. As we have shown, there is a strong emphasis on transparency of findings and stakeholder inclusion in many of the AI-IA processes investigated, both of which can be read as mechanisms to avoid the dominance of vested interests. It is not clear, however, whether they will suffice or whether independent and potentially governmental control, regulation and oversight would be required to address this concern.

A further concern is that of the functional or techno-optimist underpinnings of AI-IAs. The majority of the documents investigated started by outlining the benefits of AI, then balances these against the downsides and suggests that an AI-IA is a mechanism that will increase the likelihood that the benefits can be retained while managing risks and downsides. The techno-optimist view is that AI is fundamentally an ethically and socially good thing. In this mindset, AI-IAs are purely functional tools to ensure that AI’s benefits can unfold. This narrative pervades the AI literature and the AI policy landscape. It is, however, by no means certain that this is the only or best framing of AI in general or of specific AI technologies and applications. It may well be that the world would be better off without some particular AI technologies or applications. AI-IAs, by offering a tool to address the downsides of AI, may stifle a much broader societal debate about what future we are collectively trying to achieve and what role particular technologies should play in that future.

### Rapidly changing landscape

To conclude the discussion of AI-IAs, it is important to highlight that we are facing a rapidly changing landscape. The integrity of the methodology of a systematic literature review calls for a clear end date of data collection to ensure that the methodological rigour of the systematic approach can be preserved. The cut-off date for data inclusion is required to ensure that all sources of information are treated equally and fairly. However, this has the practical disadvantage that recent developments cannot be captured. The analysis of the data following the cut-off date takes time, as does the drafting of the paper and the review process. This means that any academic paper, by definition, is to a degree behind the curve of current and most recent publications.

This section, therefore, provides a brief overview of more recent developments and was written as part of the review process [October 2022; for a further recent overview see (Eceani et al. 2021)]. A first observation is that the development of AI-IAs continues at pace. Examples include contributions from regional and national governments, albeit all at different stages of development. (Ministerie van Algemene Zaken 2021; CAHAI 2022; Government of Canada 2022; NIST 2022). At the regional level in the Council of Europe, in February 2022 the Ad Hoc Committee on Artificial Intelligence (CAHAI) published the outcome of its work on the possible elements of a legal framework for AI, which includes discussion of non-legally binding model for an AI-IA focused on human rights, democracy, and the rule of law (CAHAI 2022). For an example at the national level, algorithmic impact assessments are mandatory in Canada under the Directive on Automated-decision Making (Government of Canada 2022). In contrast, developments in the US have come in the form of voluntary standards and policy. In August 2022, the US National Institute of Standards and Technology (NIST) published a revised draft of its recommended AI risk management framework, which includes AI-IA; the first version is due out in early 2023 (NIST 2022).

One notable aspect of these recent developments it that they provide growing levels of detail and thus seem to aim to provide increasingly applicable templates for undertaking AI-IAs. The examples cited also have a strong emphasis on human rights (rather than general ethical considerations) which may not be surprising as governments tend to visibly embrace human rights.

Similarly unsurprising, but still important, is the observation that these documents tend to strongly emphasise the legal environment in which they exist. These recent developments thus need to be interpreted in the light of developing legislation around AI which many jurisdictions around the world are currently considering. Examples of these broader developments in the field of AI would include the proposed EU AI Act, the European AI Liability Directive (European Commission 2022) or the proposal by the US government for an AI Bill of Rights (Office of Science and Technology Policy 2022) which calls for pre-deployment algorithmic impact assessments that are independent and publicly-available. A more detailed analysis of this legal landscape would call for a separate systematic review and is beyond the scope of this paper.

While various legislative agendas are progressing, the academic research on the topic also moves ahead quickly. The ethics of AI debate remains buoyant and continues to identify and analyse topics that are likely to have a bearing on how AI-IAs may be implemented (see e.g. Madaio et al. 2022). Furthermore, research on AI-IAs in the immediate sense of the term used in this article continues with recent work updating and specifying earlier contributions (see e.g. Mantelero and Esposito 2021).

What these recent developments point to are at least two issues that will be covered in the following section: Firstly, they raise the question of how a potential user of an AI-IA could determine whether a particular approach is suitable for their purposes. Secondly, the increasing emphasis on the legal but also the organisational embedding of AI-IAs calls for a more detailed understanding of their role in the broader socio-economic-technical ecosystems in which they are to be applied.

## Choosing, deploying and evaluating suitable AI-IAs

The work presented so far provides an analysis of the literature on AI-IAs. It thus fulfils the key evaluation criteria for systematic reviews which is the provision of a synthesis of the literature under investigation (Tate et al. 2015). In addition to this synthesis, it is frequently acknowledged that a systematic review should go beyond analysis and description and provided added value to the intended audience (Levy and Ellis 2006; Schwarz et al. 2007). This is often framed in terms of theory development, where the systematic review serves as the basis for identifying gaps in current theory and that insights from the review can help to address this gap. As Xiao and Watson put it, a systematic literature review “goes beyond a summary of the data and attempts to build upon the literature to create new, higher-order constructs” (Xiao and Watson 2019, p. 100). In this paper we make use of the development of higher-order constructs to provide a synthesis of our insights that can help potential users of AI-IAs to select and evaluate relevant approaches that align with their needs. This section therefore aims to transform the insights gained on the different aspects of AI-IAs discussed in the previous section into a more generic approach to measuring the impact of AI. It is aimed at an audience of academics or practitioners who are interested in understanding and applying AI-IAs and aims to develops a process to support AI-IA users in choosing and deploying an AI-IA suitable to their needs. We start by describing a generic AI-IA based on our detailed analysis and then proceed to argue that, in order to be successful, AI-IAs cannot be used in isolation but need to be seen as a part of a broader responsible AI ecosystem.

### A generic model of AI-IAs

This section aims to synthesise the insights derived from the data analysis describe in the previous sections. It is based on our insights around purpose, scope, issues, organisational context, timeframe, process and methods, transparency and challenges of AI-IAs. It is written with a possible organisational user of an AI-IA in mind, someone who has responsibility for developing or implementing an AI system in or for an organisation. Such a user may have an interest in an AI-IA as a way to mitigate risk, they may be under an obligation to consider likely outcomes or they may simply want to do the right thing. In order to navigate the multitude of existing AI-IAs and ascertain whether a particular example of such an assessment is appropriate for the use in the specific context, they will benefit from this description of a generic AI-IA which may be used to evaluate a specific AI-IA method or tool.

The first point for such a potential user to keep in mind is that an AI-IA process is triggered by a *plan* to develop or use an AI. This requires the definition of roles, most importantly for the purposes of this article, of owner(s) (person(s) responsible for) of the AI system itself, as well as AI-IA. The first task of the AI-IA owner will be the definition of the purpose of the AI, its technical description as well as the intended benefits. This conceptual work will draw on the technical expertise and the overall project management structure of the AI, which will cover similar grounds. The first unique step required by an AI-IA will then be to *answer the question whether the AI is expected to have social impact*. In most cases, one would assume this to be the case; otherwise, it would raise the question why make an investment in AI in the first place. However, it is conceivable that an AI project simply improves and optimises existing processes and hence does not have any novel social consequences. In such a case, an AI-IA could end at this early stage.

In most cases, one would assume that the development and use of an AI system will have social impact. This impact may be beneficial or detrimental to society, but it will rarely be neutral. Thus, the next step would be the *identification of the types of stakeholders and stakeholder categories that are likely to be affected* (e.g., stakeholders internal to the organisations, market participants, such as customers or suppliers, policy stakeholders or broader societal stakeholder groups including vulnerable communities). A thorough AI-IA also calls for the identification of representatives who can legitimately speak on behalf of the stakeholder groups and who should be consulted throughout all subsequent steps of the AI-IA.

A further step could be the *review of existing AI-IAs to check whether a process or template exists that is appropriate for the technology* in question and that can be adopted. If so, it can save the AI-IA owner(s) considerable time and effort, as several of the subsequent steps may already be well-defined and the existing process can be adopted. If no such appropriate AI-IA exists, then more thought must be dedicated to the following steps. Key among them is the identification of possible issues that the AI is likely to raise. These range from human rights violations and the infringement of other laws to considerations of human safety and security, environmental impact, societal issues, ethical impacts etc. In addition to identifying these issues, it is important to define metrics that can be used to capture them and assess possible changes and impacts caused by the AI.

In order to develop sensitivity to these issues and ensure they are appropriately addressed, it is desirable to integrate the AI-IA in established organisational practices, including other impact assessments, risk management and/or due diligence activities.

The steps outlined so far have set the scene for the AI-IA and defined the process. The subsequent steps can be interpreted as the *implementation of the AI-IA*. This will include the collection of data concerning possible issues and consequences, drawing on the metrics defined earlier. In light of the need for transparency, it is important to keep logs of data collection, so that later steps can draw on the evidence collected. The data and its analysis provide insights into the empirical consequences of the AI. To a large extent, the insights derived from collecting and analysing data will drive the way in which the organisation mitigates consequences of its AI implementation. Some general mitigation strategies may be defined at an earlier stage, but the detailed responses to the practical consequences of the AI implementation will often have to wait until these consequences are clearer. The understanding of practical consequences and mitigation strategies can then inform a practical action plan for the organisation.

The organisation needs to decide how much of these activities can be made public and then publish an appropriate amount of detail on the general approach, data and the action plan, so that its approach to the AI-IA and the consequences of this approach are open to scrutiny.

This concludes the first round of the AI-IA. However, there should be a *monitoring system* in place that will trigger new rounds/repetition of the AI-IA if there are either new technical and/or legal developments that may affect the insights from the first round or if unexpected consequences arise. These can then be fed back into the relevant step in the process to ensure the AI-IA remains up-to-date and relevant. The openness to re-start the AI-IA should remain in place for the duration of the use of the AI and only comes to an end once the AI is withdrawn from service or decommissioned.

**Fig. 6:**
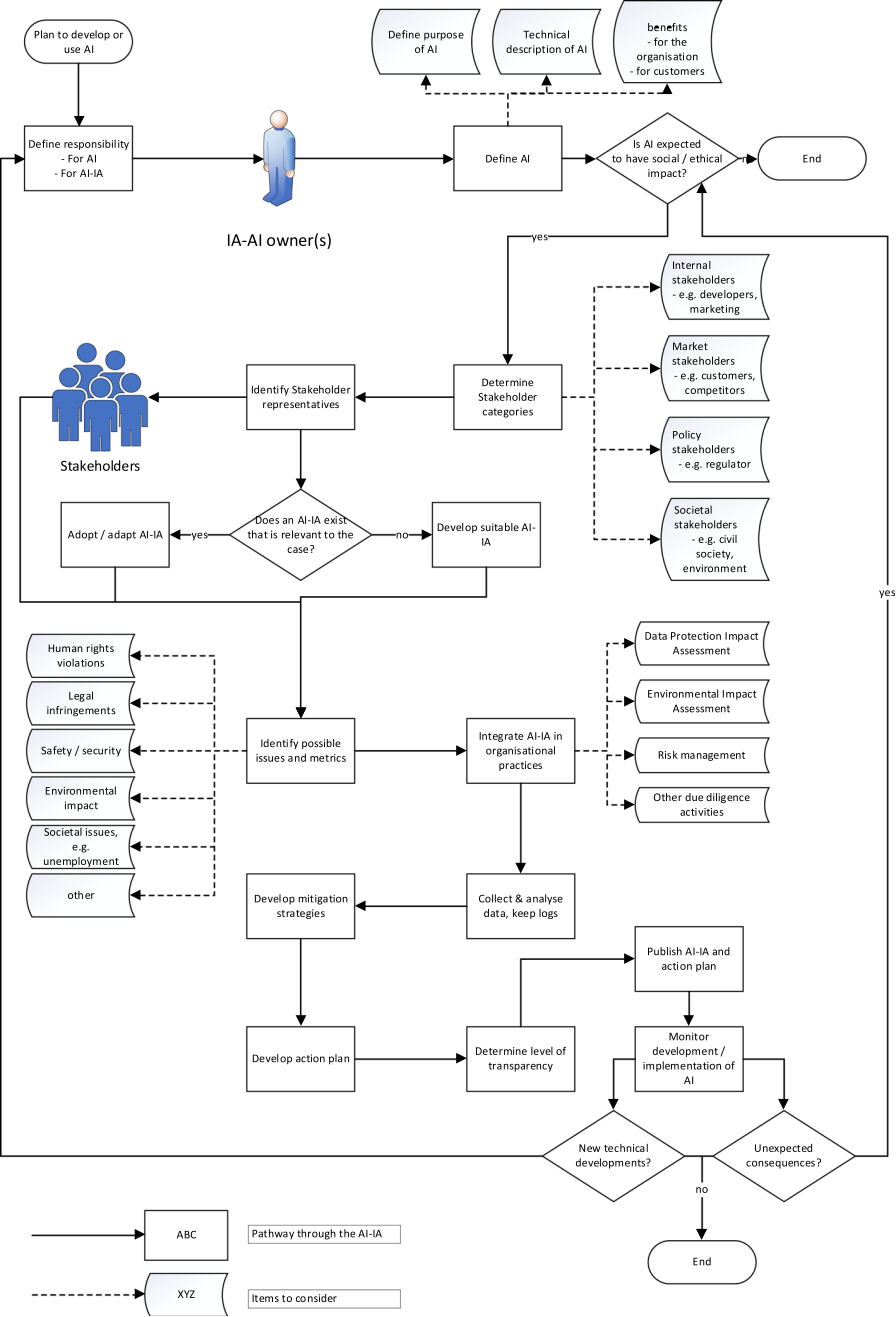
Flowchart for applying an AI-IA from an organisational perspective

This flowchart (Fig. 6) of an organisational approach to an AI-IA should not be read as a process that has to be strictly followed. The steps do not always have to follow this order. In some cases, some steps may be skipped, or additional steps taken. Its purpose here was to demonstrate that an organisation can draw on the prior work on AI-IAs, as analysed earlier in the article, to plan and implement a practical intervention to ensure that possible consequences of AI are considered early. To reiterate the point, an AI-IA is not a panacea. It cannot guarantee that all issues are identified or addressed. It should be seen as part of the organisation’s arsenal of proactively interacting with its environment to ensure that its AI-related activities are acceptable, desirable and sustainable.

Furthermore, the generic organisational perspective developed here is just one possible perspective. The logic described here could also be employed by regulators or critical observers to deconstruct an organisational approach and critically query whether it is sufficient. Moreover, AI-IAs can be considered at different levels of different systems. They could be instituted at the industry level, at the level of a region or in a technology application area. In all cases, we believe that the review of the AI-IA literature provided in this article allows a better understanding of what an AI-IA can look like, what it can reasonably achieve and where its limitations are.

### AI-IA as part of a responsible AI ecosystem

This last point about different levels of abstraction and observation highlights a further aspect of AI-IAs that is worth considering from a theoretical as well as a practical perspective. The key point is that AI-IAs need to be understood in the context of the broader AI ecosystem in which they are used. AI is not a specific technology that has clearly determined consequences in specific use cases. Instead, we propose that it is more useful to understand AI as a core aspect of a rapidly evolving ecosystem, or even as a system of ecosystems. This paper does not offer the space to develop the idea of AI as an ecosystem in detail, which we have done elsewhere (Stahl 2021; Stahl et al. 2021). However, it should be easy to see that all AI systems are complex socio-technical systems. Such systems are made up of many different components, some of which are technical artefacts, some are social artefacts, which includes individual humans, organisations and other social structures. The complexity of these systems and the idea that they interlink in many, often unpredictable, ways has given rise to the use of the metaphor of an ecosystem when talking about AI, in particular from a policy perspective (AI HLEG 2019; Digital Catapult 2020; UNESCO 2020; UK Government 2021).

The application of the metaphor of ecosystems to socio-technical systems is well established in fields such as innovation studies (Moore 1993; Adner 2006) where it is used to explain the way in which such systems grow, change and develop. It can also be used instrumentally to develop organisational strategy (Adner 2017) and position organisations in their environment. We believe that this perspective is helpful when exploring the way in which AI is developed and used. It can therefore also provide insights into the role and possible limitations of AI-IAs which form part of the broader perspective of AI ecosystems that covers ethical and social issues and that might be called responsible AI ecosystems. This perspective facilitates practical insights that can inform the choice, use and interpretation of appropriate AI-IAs.

**Fig. 7:**
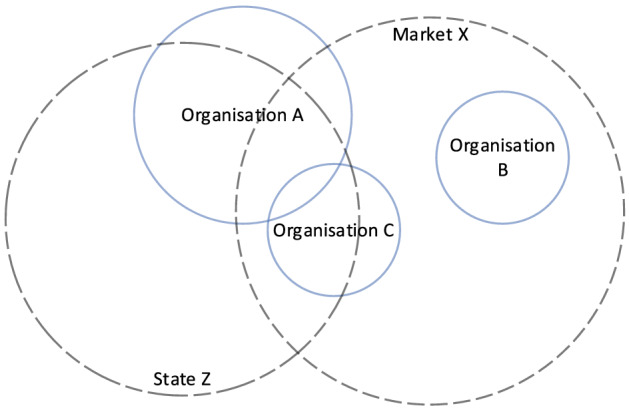
Institutional location of AI user Organisation A

We now demonstrate this way of thinking about AI using a fictitious example. In our example, Organisation A is planning to introduce an AI, say a machine learning system aimed to support radiologists in identifying breast cancer. The following eight shows that our example organisation, which itself has the character of an ecosystem, is part of other innovation ecosystems. Figure 7 shows that Organisation A partly owns Organisation C but also has competitors, in this case, Organisation B. The market in question is Market X, the market of radiological diagnostics systems. This is not the only market in which Organisation A is active. At the same time, Organisation A is partly located in the territory of State Z which drives the regulatory requirements of the market in Z’s jurisdiction.

In this context, the aim of an AI-IA is not so much the precise prediction of the consequences of use, but the initiation of a reflection process that aims to achieve acceptable, desirable and sustainable outcomes. In adopting such a systems-oriented view of AI, possible users of an AI-IA need to realise that an AI-IA is not a stand-alone activity, but that it forms part of an array of interventions that share the aim of ensuring beneficial consequences of AI development and use. Such other interventions range from international/national regulation, certification schemes (AIEI Group 2020), standards (IEEE Computer Society 2021), professional guidance (Brinkman et al. 2017), ethics frameworks (AI HLEG 2019), development methodologies (Martin and Makoundou 2017; Fjeld et al. 2020) etc. Similarly, the actors involved in AI ecosystems are multiple and overlapping, spanning across the public and private sectors and ranging from international organisations such as the UN or OECD to nation states, regulators, developers, system deployers, users and civil society.

This example is typical for many organisations planning to use AI. The purpose of introducing it here is to underline the nature of overlapping innovation ecosystems that influence AI decision-making and thus the possible use of AI-IAs. Similar diagrams could be drawn for the technology in question or for the application area. Its main purpose is to underline the character of AI as a socio-technical system embedded within other systems that precludes simple and straightforward interventions.

The benefit of applying the ecosystem concept to AI for the purposes of this paper is that it demonstrates that AI-IAs are not fixed tools and that it is not a simple matter of applying a standard format to a new technology with a reasonable expectation that this will solve the social and ethical problems associated with AI. Instead, they should be seen as part of numerous ongoing processes where many technical, human and organisational actors have roles to play in reflecting on the technology, its possible consequences and ways to deal with these. This context is important to consider when working with an AI-IA as proposed in the previous section and Fig. 6 as it shows that the context to consider when undertaking an AI-IA may vary substantially depending on the AI ecosystem that it is meant to be applied to. Clarifying the link of the AI-IA to other components that are meant to render the AI ecosystem responsible, such as standards, methodologies, assurance mechanisms etc. will render it more likely that the AI-IA is impactful and achieves the intended aim of highlighting issues and facilitating their resolution.

## Conclusion

This article offers the first systematic review of AI-IAs. In light of growing interest in the ethics and regulation of AI, it can be expected that AI-IAs will play an important role in future AI governance. The article therefore will be of interest to researchers working on AI ethics and AI policy. It also makes a practical contribution that is relevant to both policymakers who are considering how to implement AI policies and organisations interested in using an AI-IA to better understand and reflect on their technologies or aiming to broaden their risk management processes.

As any research, this article has limitations. We set out to undertake a systematic review of AI-IAs. However, the nature of these documents rendered it difficult to arrive at an incontrovertible population of documents. We believe that our multi-pronged search strategy allowed us to identify all or, at least, the most relevant AI-IAs. We cannot prove this and new AI-IAs will have become available since we undertook the search in the summer of 2021. In addition, the conceptual fuzziness of AI means that it is challenging at best to precisely delineate the inclusion and exclusion criteria. Due to our search strategies, our sample included some documents that focus on closely related questions such as data ethics (UK Governmental Digital Service 2020) and were found to fall within our definition of AI-IAs, but we concede that different interpretations are possible, leading to a different population of AI-IAs. It is unlikely, however, that the inclusion of additional AI-IAs or the removal of parts of the documents we analysed would fundamentally alter our findings.

This article should provide a sound basis for the next step in developing AI-IAs. The documents analysed include several well-researched, mature and reflected examples that can be implemented by organisations. What seems to be missing now is a more comprehensive overview of their role in the AI ecosystem. We have shown that there is much attention to other types of impact assessments, calls for the coordination with such impact assessments, consideration of the integration of AI-IAs into other organisational processes such as risk management as well as numerous references to relevant regulation.AI-IAs need to be understood in this broader context.

At present, there is little guidance on the role of AI-IAs in the broader context of the AI innovation ecosystems. This makes it challenging for organisations planning to use AI to identify the most appropriate AI-IA for their specific needs. This contributes to the challenge of evaluating whether a particular AI-IA is fit for purpose and whether an organisational application of it can or will have the desired outcome.

Some of these problems are likely to be temporary and upcoming legislation, regulation, professional guidance and case law will make the role of AI-IAs in their ecosystems clearer. Meanwhile, there is need for research to better understand the impact of AI-IAs. They are typically framed in terms of the benefits they offer for individuals, organisations and society as a whole. It is currently unclear whether the application of an AI-IA actually leads to the promised benefits and how this could be measured. Such research is urgently needed to ensure that AI-IAs can contribute to addressing the ethical and social consequences of AI use, while simultaneously not overloading them with unachievable expectations. We hope that this research has provided a robust evidence base for such further research and thereby contributes to the overall aim of ensuring that AI contributes to human flourishing.

## Footnotes

1 The final set of AI-IAs that were fully analysed are publicly available via a Zotero group library (https://www.zotero.org/groups/4042832/ai_impact_assessments).
